# The impacts of host community composition, clustering and vector host choice on zoonotic mosquito-borne virus transmission

**DOI:** 10.1371/journal.pntd.0014758

**Published:** 2026-10-22

**Authors:** Jennifer S. Lord, Michael B. Bonsall, Tijani A. Sulaimon

**Affiliations:** 1 Department of Vector Biology, Liverpool School of Tropical Medicine, Liverpool, United Kingdom; 2 Department of Biology, University of Oxford, Oxford, United Kingdom; Washington State University, UNITED STATES OF AMERICA

## Abstract

Zoonotic mosquito-borne viruses, including West Nile, Rift Valley fever and Japanese encephalitis viruses, present a substantial public health burden in some countries, with transmission involving complex interactions between multiple vector and host species, making control difficult. While host community composition is known to affect transmission, the role of host community structure coupled with mosquito aggregation behaviour remains poorly understood. Motivated by furthering understanding of potential Japanese encephalitis virus transmission routes and persistence in contexts where cattle – dead-end hosts – are dominant in the community, we developed a patch model in which host distributions and the resulting mosquito biting allocation among patches are fixed within each simulation. This framework represents mosquito host-seeking across two scales: long-range aggregation toward host-dense patches and short-range host species choice determined by local host availability and innate vector preference. Using this model, assuming that vectors prefer dead-end hosts (choosing them 90% of the time if given an equal choice of other hosts), we explored the potential effect of host clustering, patch fragmentation, and the strength of mosquito aggregation on the basic reproduction number. Our results show that identical host communities can yield very different transmission outcomes depending on how they are distributed and the level of mosquito aggregation. Under circumstances where dead-end hosts are preferred by vectors, if competent hosts are clustered and mosquitoes aggregate strongly, transmission may be amplified compared with uniformly distributed hosts and vectors. If dead-end hosts are clustered, transmission may be diluted as mosquitoes aggregate in patches where dead-end hosts are dominant in the host community. Fragmentation effects are context-dependent: when competent hosts are clustered and mosquitoes aggregate, increasing patches increases transmission. When dead-end hosts are clustered, transmission decreases under strong mosquito aggregation with increasing number of patches. These findings highlight that variations in the local spatial context of host-vector interactions can lead to variations in disease risk. Locally-informed surveillance and targeted interventions may therefore be important in attempts to prevent outbreaks of zoonotic mosquito-borne viruses.

## Author summary

Zoonotic viruses spread by mosquitoes, such as West Nile, Japanese encephalitis, and Rift Valley fever viruses, pose threats to human and animal health. These pathogens can circulate among different host species, some that amplify transmission (competent hosts) and others that do not (dead-end hosts). Across a landscape, mosquitoes may aggregate in areas of higher host density and may then choose a host depending on their innate preference and relative host availability. Therefore, processes affecting vector host choice operate at two different spatial scales.

We developed a mathematical model to explore how clustering of hosts and mosquitoes’ tendency to aggregate in areas supporting high host density affect virus transmission. Our simulations show that grouping competent hosts can increase transmission if mosquitoes strongly cluster there. Conversely, clustering dead-end hosts reduces transmission by drawing mosquitoes away from competent ones. Landscape fragmentation, like dividing areas into more patches, has mixed effects depending on host grouping and mosquito behaviour.

Our work shows that the same community of animals can pose a very different disease risk depending entirely on their spatial arrangement, highlighting the need to consider local conditions in vector surveillance and control.

## Introduction

There are at least 21 mosquito-borne viruses that cause disease in humans but for which humans are a ‘dead-end’ host and require other vertebrates to maintain transmission [1]. These zoonotic arboviruses include Japanese encephalitis virus (JEV), Rift Valley fever virus (RVFV) and West Nile virus (WNV). Zoonotic arbovirus transmission often involves multiple vector species that feed on both humans and other vertebrates, leading to communities of host and vector species involved in pathogen transmission and persistence. For example, the transmission of JEV can involve cycles between multiple mosquito species, including *Culex vishnui*, *Culex tritaeniorhynchus*, *Culex gelidus*, *Armigeres subalbatus*, *Mansonia* spp and vertebrate hosts, including pigs and a variety of birds [2–6]. Similarly, RVFV is transmitted between multiple *Culex* and *Aedes* species and ungulates, including wildlife, cattle, goats and sheep [7–9] and West Nile virus is also transmitted between multiple mosquito and bird species [10–12]. The transmission dynamics of zoonotic arboviruses are therefore influenced by complex relationships between the vector community and vector species’ behaviour, host community composition, and the spatial structure of these interactions [13].

For any vector-borne disease, vector host choice plays a key role in determining pathogen transmission risk by influencing patterns of vector-host contact rates [14]. Vector species often have innate preference for certain host species, but can behave opportunistically, especially when preferred hosts are scarce [15]. Vector host choice therefore depends not only on innate host preference but also on relative host availability. The outcome of the interactions between vector behaviour, host community composition and spatial structuring on vector host choice is especially important when host communities comprise species of varying competence for the pathogen in question, as is often the case for zoonotic mosquito-borne viruses.

For over 100 years, vector-borne disease researchers have been interested in vector host choice (as summarised in [16]). In particular, the possibility of using non-human animals to divert vectors of human-infective pathogens away from humans; termed ‘zooprophylaxis’, has been a primary motivation. However, the potential for complexities within local ecological contexts to determine either dilution or amplification of transmission have also long been acknowledged. For example, Sota and Mogi [17] demonstrated conditions where the presence of domesticated animals could increase malaria infection through increasing mosquito densities and therefore human biting rates, overcoming any reductions gained through diverting bites away from humans.

The ideas and evidence of the drivers of zooprophylaxis, or the opposite effect - zoopotentiation, may be placed within a broader framing of the effects of species diversity on disease risk. Keesing *et. al.* [18] outlined the mechanisms by which host species diversity may either dilute or increase pathogen transmission risk, by affecting encounter rates, density of susceptible hosts, recovery rates and death rates. Further to this, Miller and Huppert [19] used a general modelling framework to demonstrate that higher host diversity can lead to pathogen amplification if vector preference for highly competent host species is sufficiently strong to overcome any losses in transmission to less competent species. Conversely, dilution can occur when vectors prefer less competent hosts or display no specific host preference. These generalisations have been illustrated in specific zoonotic mosquito-borne virus systems. For example, Simpson *et. al.* [20] demonstrated, for WNV in the USA, that *Culex pipiens* preference for American robins induced contact heterogeneity between hosts and vectors which was a key mediator in transmission intensity and timing. Further complexities can also play out in specific systems. For example, by using a long-term data series of plague dynamics in prairie dogs, Stapp *et. al.* [21] demonstrated that the presence of alternative host species can sustain transmission through vector host-switching when prairie dogs died of disease.

This body of knowledge has furthered our understanding of the impacts of heterogeneity in vector host choice on transmission in multi-host systems. Our own questions in this area are motivated by JEV in particular. We recently demonstrated that vector fidelity, defined as the tendency of vectors to feed on the same host species from which a first meal was taken, creates non-homogeneous mixing between host and vector populations, and therefore can affect transmission. We showed that sufficient fidelity could allow JEV to persist in areas like Bangladesh, where pigs, though highly competent hosts, are outnumbered by cattle, which are dead-end hosts for JEV [22]. In Bangladesh, birds may play a role in transmission, but this remains to be determined [23]. In addition to fidelity, which involves sequential conditioning, there are other mechanisms by which JEV may be maintained, including spatial arrangements of dead-end hosts relative to competent hosts. We are, however, not aware of a framework for considering questions of host distribution, grounded in an understanding of the biological processes affecting vector host choice. Here, we propose to view mosquito vector host choice as a process that operates at two spatial scales. At the long-range, mosquitoes use cues such as carbon dioxide and potentially visual stimuli, to locate patches of hosts. Once within a patch, at the short-range scale mosquitoes use cues such as skin odourants, heat, and visual stimuli to select specific hosts [24–26]. In this theoretical modelling study, we ask how structuring in the host community and variation in community composition between patches (e.g., households) may influence transmission under the following scenarios: i) when mosquitoes are equally likely to visit any patch and are not influenced by the numbers of hosts present; ii) when mosquitoes are more likely to visit patches with greater numbers of hosts. We specifically address these questions assuming dead-end host species are preferred by vectors.

Our work here focuses on vector host preference and aggregation, highlighting another way in which transmission may be maintained in systems where dead-end hosts are dominant and preferred by the most abundant vector species. While empirical data often vary spatially, and some zoonotic mosquito-borne virus models, such as those of Simpson *et. al.* [27] and Ladreyt *et. al.* [28], account for host communities and vector feeding preferences, they often neglect the potential role of structuring in host communities.

## Methods

### Vector-host interactions

We make the simplifying assumption that at the ‘long-range’ scale, vectors cannot differentiate between preferred and non-preferred host species, and that this requires cues only available at the ‘short-range’. We first discuss processes at the long-range and then deal with assumptions about short-range host-selection.

### Aggregation of biting vectors (long-range)

With respect to long-range host seeking by mosquitoes, Gillies [29] estimated that the range of attraction towards a CO_2_ source increased linearly with the amount of CO_2_ emitted, from *c.* 10 metres at 300 ml/min to *c.* 30 metres at 1000 ml/min. Therefore, an increase in host density in a given location - herein ‘patch’, and as a consequence the amount of CO_2_ released, may increase the number of host-seeking vectors attracted to that patch. For at least some mosquito species, this increase in abundance can be more than linear [30,31]. Lord *et. al.* [31], in northwest Bangladesh, showed that for each additional cow present in a household, there was an approximate two-fold increase in the number of *Culex tritaeniorhynchus* and approximately 1.5 to 1.7–fold increase of other common species. However, even if mosquitoes are attracted to high host density patches, assuming aggregation, patches with low host density could still attract some mosquitoes due to interference between mosquitoes in high-density patches or spatial arrangement with respect to larviposition and resulting breeding sites. Evidence for interference between mosquitoes includes increased host defensive behaviour, in addition to a decline in the proportion of those that are blood fed, with increasing density of host-seeking mosquitoes in the vicinity of hosts [32]. We define interference as the prevention or limiting access to a patch [33,34].

To quantify the effects of non-random contact due to spatial variation in host density and community composition, we assumed that hosts are distributed across *n* discrete patches, with mosquitoes having equal potential access to all patches (i.e., within flight range). This simplifying assumption was based on distances between households being relatively small compared with estimates of JEV vector dispersal. The average pairwise distance between households in a village in northwest Bangladesh is *c.* 250 m and the maximum distance between households within a village frequently less than 1 km. *Culex tritaeniorhynchus* mosquitoes can disperse at least 0.65 km 48 hrs after emergence [35] and Wada *et. al.* [36] recorded an initial dispersal on day one of release of between 0.9 and 5.1 km and a mean dispersal distance of 1 km over the seven day period.

With these assumptions, we considered three scenarios: (1) mosquitoes are equally distributed between patches (1/*n*); (2) mosquitoes aggregate as a function of the total host density or CO_2_ emission in each patch but also an opposing factor of interference; or (3) mosquitoes aggregate predominantly as a function of the total host density or CO_2_ emission in each patch.

This host-dependent distribution of mosquitoes may be thought of as similar to how predators may distribute themselves in response to prey density. Early ecological models suggested that predators concentrate in areas of high prey densities [37]. However, aggregation can lead to interference among predators, reducing foraging efficiency and potentially causing them to move away from high-density patches [38,39]. Initially, Hassell and May [33] formalised this idea for host-parasitoid systems by introducing an aggregative response that depends on both prey density and an aggregation constant ($1/m_{p}$). Sutherland [34] later adapted this framework to incorporate interference ($m_{p}$) more explicitly, which we use here to describe the level of mosquito aggregation - how mosquitoes respond to how hosts are distributed:


$$\begin{array}{c} \delta_{v,i}=\frac{a_{x,i}^{1/m_{p}}}{\sum_{j}a_{h,j}^{1/m_{p}}}, \end{array}$$ (1)

where $\delta_{v,i}$ is the proportion of mosquitoes in the *i*th patch, $a_{h,i}$ is the proportion of all hosts (or the relative CO_2_ emission) from the *i*th patch, and the parameter $m_{p}\in (0,1]$ measures the strength of interference [34]. The term $1/m_{p}$ corresponds to the aggregation constant from Hassell and May [33]. Mosquito aggregation is created as $m_{p}$ decreases, mid-range values for $m_{p}$ will create aggregation with moderate interference (scenario 2), as the exponent $1/m_{p}$ (aggregation) becomes very large, aggregation becomes strong (scenario 3). Given that $a_{h,i}\leq 1$, even small differences in host density are greatly amplified, making patches with high host density more attractive and leading mosquitoes to aggregate more strongly in these patches.

Although mosquitoes are sometimes assumed to be uniformly distributed across space ($\delta_{v,i}=1/n$), there is no biological basis for this assumption. Analysis of household mosquito catch data from Bangladesh [31], using the Sutherland aggregation model (Equation 1), suggests biological evidence for aggregation with interference (fitted value $m_{p}=0.36$; Fig 1, S1 File, see S1 File for equation fit to the data) - in reality mosquitoes tend to aggregate disproportionately in patches with high host densities and that some level of interference influences their spatial distribution. We show this as biological evidence and motivation for the assumptions made in our modelling; varying values of $m_{p}$ to highlight the potential effects of vector aggregation, rather than modelling using this fixed fitted value, which may be different across species and contexts.

**Fig 1:**
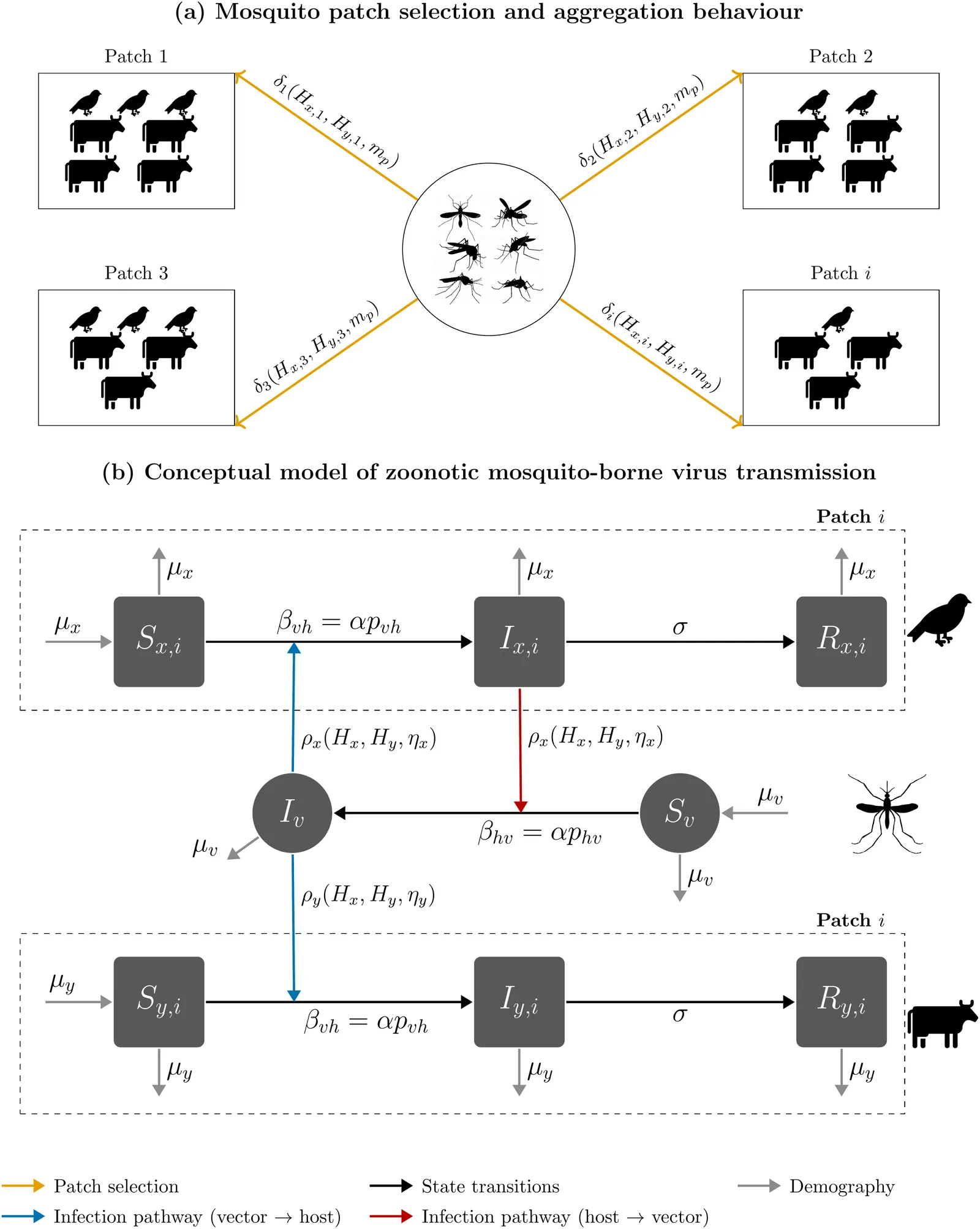
a) Mosquito aggregation and patch selection. Patches are fixed with no host movement, and mosquitoes have equal access to all patches. Mosquitoes are either uniformly distributed across patches (δ=1/n where *n* is the number of patches) or aggregated based on total host density in each patch and level of interference $m_{p}$. **b)** Compartmental model. The flow diagram illustrates hypothetical zoonotic mosquito-borne virus transmission within a patch comprising two host species (competent hosts, birds and dead-end hosts, cows) and free-roaming adult female mosquitoes (*v*). Susceptible mosquitoes can become infected by biting infected birds ($I_{x,i}$) and may die in any compartment. Birds can become infected when bitten by infected mosquitoes and subsequently recover. The proportion of mosquito bites on birds and cows, determined by mosquito preference and host availability, is $\rho_{x}$ and $\rho_{y}=(1-\rho_{x})$, respectively. Hosts are born susceptible and may die in any compartment. Solid black arrows indicate state transitions; red dashed arrows represent host-vector infection interactions. Cow, bird, and mosquito images obtained from https://www.phylopic.org, licensed under CC0 1.0.

### Host species choice (short-range)

We assume that once within a patch, mosquitoes are within an equidistant short-range of all individuals of all host species present in that patch. In the absence of any preference for a given host species, vector host species choice will, in this scenario, be dependent entirely on the relative densities of each host species present, thus the proportion of blood meals on competent host species *x*, in a two host species community is simply:


$$\begin{array}{c} \rho_{x}=\frac{H_{x}}{H_{x}+H_{y}} \end{array}$$ (2)

where $H_{x}$ and $H_{y}$ are numbers of competent (*x*) and dead-end host species (*y*) respectively. This model would reflect host choice by an opportunistic vector species. Vectors can, however, show preference for certain host species [17,27,40,41]. Vector preference for competent host species ($\eta_{x}$) can be added in to Equation 2 to obtain:


$$\begin{array}{c} \rho_{x}=\frac{H_{x}\eta_{x}}{H_{x}\eta_{x}+(1-\eta_{x})H_{y}}, \end{array}$$ (3)

as per Sota and Mogi [17], assuming $\eta_{x}$ is the probability an individual vector chooses host *x* over *y* when both are equally available.

### Transmission models incorporating vector host choice

A vector-borne disease model can be formulated where host species *x* ($S_{x},I_{x},R_{x}$) are competent hosts (total individuals $H_{x}$) that contribute to arbovirus transmission, while host species *y* (total individuals $H_{y}$) are non-competent hosts that do not support onward transmission. The formulation follows Fig 1 in which susceptible ($S_{v}$) or infected ($I_{v}$) mosquitoes can bite birds (assumed competent, host species *x*) and cows (assumed dead-end, host species *y*) at a rate α. Susceptible mosquito ($S_{v}$) bites on infected birds ($I_{x}$) can lead to infection with a probability $p_{hv}$ and infected mosquitoes ($I_{v}$) can pass the infection to susceptible birds ($S_{x}$) with probability $p_{vh}$. Infected mosquito ($I_{v}$) bites on cows ($H_{y}$) do not cause onward transmission.

For this pathogen transmission problem, we model the changes in the number of competent and susceptible hosts and mosquitoes. The ODEs for this are then:


$$\begin{array}{cc} \frac{dS_{x}}{dt} & =\mu_{x}H_{x}-\beta_{vh}\rho_{x}I_{v}\frac{S_{x}}{H_{x}}-\mu_{x}S_{x}\\ \frac{dI_{x}}{dt} & =\beta_{vh}\rho_{x}I_{v}\frac{S_{x}}{H_{x}}-(\mu_{x}+\sigma )I_{x}\\ \frac{dR_{x}}{dt} & =\sigma I_{x}-\mu_{x}R_{x}\\ \frac{dS_{v}}{dt} & =\mu_{v}N_{v}-\beta_{hv}\rho_{x}\frac{I_{x}}{H_{x}}S_{v}-\mu_{v}S_{v}\\ \frac{dI_{v}}{dt} & =\beta_{hv}\rho_{x}\frac{I_{x}}{H_{x}}S_{v}-\mu_{v}I_{v} \end{array}$$ (4)

where mosquito births are equal to deaths, similar for hosts, $H_{x}$ is the total number of host species *x* ($S_{x}+I_{x}+R_{x}$) and $N_{v}$ is the total number of mosquitoes ($S_{v}+I_{v}$). The biting rate and the probability of infection given a bite is represented by $\beta_{vh}$ for mosquitoes to hosts and $\beta_{hv}$ for hosts to mosquitoes, and $\mu_{x}$ and $\mu_{v}$ are density-independent mortality rates for competent host species and mosquitoes, respectively. We did not include dead-end hosts in our dynamic model as they do not contribute to transmission. If the framework was adapted to inform and integrate data from field studies, then dead-end hosts could be incorporated if they were to be used, for example for estimates of the force of infection using IgG antibodies as an indicator of past exposure. Like other vector-borne disease models, our model assumes frequency-dependent transmission; the transmission rate does not depend on the density of the competent host population as we assume mosquitoes bite at a fixed rate determined by the length of the gonotrophic cycle. Importantly, here we model the proportion of bloodmeals on competent hosts ($\rho_{x}$) as a function of the competent and non-competent host densities and underlying vector preference for competent hosts ($\eta_{x}$) (Equation 3).

### Extending to *n*n patches and incorporating mosquito aggregation

We extended the model in Equation 4 to compare transmission in scenarios where the host community is spread over *n* patches and either: i) the mosquito population is evenly spread across patches (Equation 5), where the numbers in each mosquito compartment are simply divided by the number of patches to distribute mosquitoes over hosts. Mosquitoes can then feed on any host in any patch with equal likelihood; or ii) mosquitoes aggregate in patches where host density is higher; δ is a (mathematical) vector of proportions following Equation 1 for which $a_{i}$ of the $i^{th}$ patch is equivalent to the sum of competent and dead-end hosts present in the $i^{th}$ patch ($H_{x,i}+H_{y,i}$) divided by the total hosts in the system $H_{x}+H_{y}$. For a given host distribution, δ is constant in time; the model therefore represents the expected allocation of mosquito biting among patches rather than explicit movement flows. Therefore our model does not explicitly involve the movement of mosquitoes between patches, because at any time point, mosquitoes have equal access to each patch and the allocation is not dependent on previous patches visited.


$$\begin{array}{cc} \frac{dS_{x,i}}{dt} & =\mu_{x}H_{x,i}-\beta_{vh}\rho_{x}(\delta_{v,i}I_{v})\frac{S_{x,i}}{H_{x,i}}-\mu_{x}S_{x,i}\\ \frac{dI_{x,i}}{dt} & =\beta_{vh}\rho_{x}(\delta_{v,i}I_{v})\frac{S_{x,i}}{H_{x,i}}-(\mu_{x}+\sigma )I_{x,i}\\ \frac{dR_{x,i}}{dt} & =\sigma I_{x,i}-\mu_{x}R_{x,i}\\ \frac{dS_{v}}{dt} & =\mu_{v}N_{v}-\beta_{hv}\rho_{x}\sum_{i}^{n}\frac{I_{x,i}}{H_{x,i}}(\delta_{v,i}S_{v})-\mu_{v}S_{v}\\ \frac{dI_{v}}{dt} & =\beta_{hv}\rho_{x}\sum_{i}^{n}\frac{I_{x,i}}{H_{x,i}}(\delta_{v,i}S_{v})-\mu_{v}I_{v} \end{array}$$ (5)

In Equation 5, *S_x,i_* $I_{x,i}$, $R_{x,i}$ are (mathematical) vectors of length *n* patches. For mosquito host choice, values of ρ are calculated for each patch depending on the number of competent and dead-end hosts present there. From the full model in Equation 5, we derived an expression for the basic reproduction number (*R*_0_) under the assumption of an infection-free equilibrium, the state where the pathogen is entirely absent from the population (S1 File).

### Effects of host distribution and mosquito aggregation on mosquito host choice

We first modelled the effect of mosquito aggregation (Equation 1) on host choice and the overall proportion of blood meals on competent hosts, under different distributions of hosts across discrete patches. We did this by comparing 12 scenarios relative to a baseline. The baseline scenario assumes hosts are evenly distributed across patches and that there is no mosquito aggregation, so that the mosquito population is also evenly distributed. This is the same as assuming an homogenous host and mosquito population in a single-patch system; an assumption made in many transmission models [42]. The 12 scenarios combined: i) competent hosts weakly clustered across patches; ii) competent hosts strongly clustered across patches; iii) dead-end hosts weakly clustered across patches; and iv) dead-end hosts strongly clustered across patches (Fig 2A), with either no, weak ($m_{p}=0.9$) or strong ($m_{p}=0.1$) mosquito aggregation (Fig 2B).

**Fig 2:**
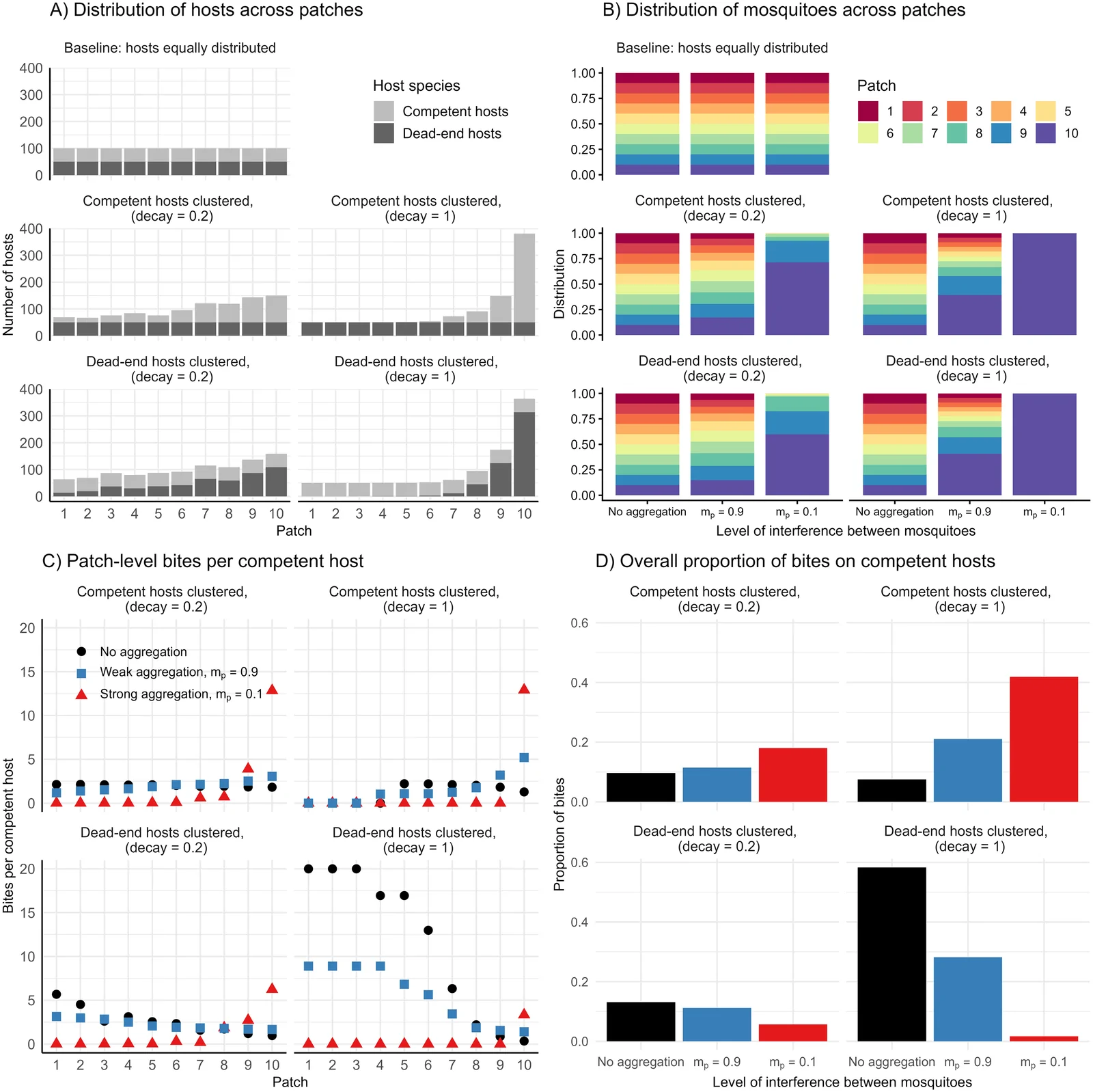
Host clustering and mosquito aggregation influence mosquito host choice. We demonstrate the effects of different distributions of hosts across patches, and levels of mosquito aggregation, on the overall proportion of bites on competent hosts. **A)** Example distributions of competent and dead-end hosts across 10 patches under different scenarios using multinomial draws. Top left: baseline - both competent and dead-end hosts equally distributed across patches. Middle row: competent hosts either strongly or weakly clustered in a few patches. Bottom row: dead-end hosts either strongly or weakly clustered. Note, host clustering was generated using multinomial draws. **B)** Mosquito distributions resulting from host clustering as shown in A, under different levels of mosquito aggregation: i) no aggregation; ii) weak aggregation (strong interference, $m_{p}=0.9$); and strong aggregation (weak interference, $m_{p}=0.1$). **C)** The total bites on competent hosts in each patch according to the different scenarios presented in A and **B. D)** Consequences for the overall proportion of blood meals on competent hosts across all patches (using a single multinomial draw).

Different host distributions were modelled using an exponential decay function to represent varying levels of clustering. First, unnormalised weights were assigned to each patch as $w_{i}=\exp(-\lambda i)$, for i=1,...,n, and probabilities were then calculated as $p_{i}=w_{i}/\sum_{j=1}^{n}w_{j}$. These probabilities were used in a multinomial distribution to allocate hosts across patches. Therefore, low values of decay (λ) would lead to weak clustering, and high values of decay (λ) to strong clustering in a few patches. Note that while host allocation across patches is stochastic (multinomial draws), the total number of hosts in each category is conserved across replicates; therefore, variability arises only in the distribution across patches, not in overall community composition.

For each scenario, the number of patches was fixed to 10 to aid visualisation, the total number of competent hosts ($H_{x}$) fixed to 500 and mosquitoes ($N_{m}$) 10,000, giving an average mosquito:host ratio of 20 (Table 1). Mosquito preference for competent hosts ($\eta_{x}$) was fixed at 0.1 (Table 1), focusing on circumstances where dead-end hosts may be preferred. Using these parameter values, for each scenario, for each patch, we calculated the number of mosquito bites per competent host and from this, for each scenario, the overall proportion of bites on competent hosts.

**Table 1:** Fixed variables and model parameters. The “Value (Range)” column shows baseline values used in simulations, with the range explored in sensitivity analyses shown in parentheses. Sources indicate whether the value is assumed or based on literature. Parameter values and ranges were selected to be broadly applicable to zoonotic mosquito-borne virus transmission with avian reservoir hosts.

Symbol	Definition	Value (Range)	Source
**Biological parameters**			
$\mu_{v}$	Mosquito mortality rate (per day)	1/20 (1/30, 1/10)	[43]†
$\mu_{x}$	Host mortality rate (per day)	1/365 (1/1825, 4/365)	Assumed ‡
α	Mosquito biting rate (per day)	1/3 (1/5, 1)	[44]
$p_{hv}$	Probability of infection: host to mosquito	0.5 (0, 1)	Assumed
$p_{vh}$	Probability of infection: mosquito to host	0.5 (0, 1)	Assumed
σ	Host recovery rate (per day)	1/5 (1/14, 1/4)	[45]
$\eta_{x}$	Mosquito preference for competent hosts	0.1, 0.9 (0, 1)	[22] *
**System variables and spatial parameters**			
$H_{x}$	Total number of competent hosts	500	Assumed
$H_{y}/H_{x}$	Dead-end to competent host ratio	1 [1, 100]	Assumed
$N_{m}/H_{x}$	Mosquito-to-competent-host ratio	20 (1, 100)	[31]
$n_{p}$	Number of patches	10, 20 [1, 50]	Assumed
$m_{p}$	Strength of interference	(0, 1]	[33,34]
λ	Exponential decay of host distribution	0.2, 1 (0, 2)	Assumed

† Showed that under insectary conditions, the lifespan of female *Cx. tritaeniorhynchus* ranged from 18.2 to 29.8 days.

‡ Range corresponds to avian host lifespans of approximately 3 months to 5 years.

* JEV vector host preference for birds not known, but see supplementary material of [22], estimates range from 0.05 to 0.22 of preference for pigs when given the choice of either pigs or cattle.

### Model analysis: implications for *R*_0_ and epidemic dynamics

Having demonstrated the effects of varying host distributions and mosquito aggregation on the proportion of blood meals on competent hosts, we used the model to quantify effects on *R*_0_ and epidemic dynamics, under different levels of host and mosquito aggregation. We again compared variations to the baseline described in the previous section (Table 2).

**Table 2:** Summary of model analyses.

Analysis	Parameters varied	Parameters fixed	Sub-question
Host ratio (Fig 3A)	Dead-end:competent host ratio, host clustering, mosquito aggregation	20 patches, $\eta_{x}=0.1$	How does increasing dead-end hosts affect *R*_0_ under different clustering scenarios?
Fragmentation effect (Fig 3B)	Number of patches, host clustering, mosquito aggregation	Host ratio = 1, $\eta_{x}=0.1$	How does patch number affect *R*_0_ under different host clustering and mosquito aggregation scenarios?
Dynamics (Fig 3C)	Host clustering, mosquito aggregation	20 patches, host ratio = 1, $\eta_{x}=0.1$	How do host clustering and mosquito aggregation affect epidemic timing and magnitude?
Sensitivity (Fig 4)	All parameters	—	Which parameters most strongly influence *R*_0_?

We first assessed how transmission changes with dead-end:competent host ratio, when there is mosquito aggregation. To do this, we fixed the number of patches at 20, and estimated *R*_0_ under the 12 scenarios combining: i) no (baseline), weak ($m_{p}=0.9$) and strong ($m_{p}=0.1$) mosquito aggregation; ii) uniform (baseline), weak (λ=0.2) and strong (λ=1) host clustering.

In scenarios where the host ratio varied, the number of patches was fixed at 20. The number of patches over which a fixed host density is distributed may, however, also affect relative differences between scenarios assuming no, weak or strong mosquito aggregation. For example, as the number of patches increase, if competent hosts are clustered, there will be a greater number of patches containing only dead-end hosts (S2 File, Fig 1). We therefore also simulated the effects of distributing hosts over an increasing number of patches. When the number of patches varied, the host ratio was fixed at 1. In both scenarios, we measured the change in *R*_0_, using the scenario where hosts and mosquitoes are equally distributed as the baseline ($R_{0}^{baseline}$) for comparison. Change in *R*_0_ is defined as:


$$\begin{array}{c} \Delta R_{0}=R_{0}-R_{0}^{baseline}. \end{array}$$

Finally, we simulated the model using the parameter values listed in Table 1 to compare how different host distributions and varying levels of mosquito aggregation could influence the occurrence, timing, and magnitude of epidemic outbreaks. Our focus was on scenarios where the dead-end host is preferred by vectors, but we also ran the simulations assuming a strong vector host preference for competent hosts (η=0.9), the results of which we present in S2 File Fig 2. All reported results represent the mean with confidence intervals over 100 stochastic replicates of host distributions.

### Sensitivity and uncertainty analysis

To explore the influence of each parameter on *R*_0_, we performed sensitivity and uncertainty analyses [46]. Using Latin hypercube sampling (LHS), we generated 1000 sets of parameters within the ranges specified in Table 1. Each set was used to calculate *R*_0_. For uncertainty analysis, we plot the distributions of each parameter resulting in *R*_0_ > 1 or *R*_0_ < 1 to identify the parameters associated with the risk of an outbreak (*R*_0_ > 1) [47]. Sensitivity of *R*_0_ to variability in each parameter was assessed by calculating partial rank correlation coefficients (PRCC) to assess how changes in each parameter affect *R*_0_ [48,49]. This analysis was implemented for different scenarios of host distributions and was performed using the *Sensitivity package* in R [50]. A summary of all the analyses performed are shown in Table 2.

## Results

### Host and mosquito distribution patterns affect the proportion of blood meals on competent hosts

The distribution of hosts and mosquitoes across patches influences bite patterns at both the patch and population levels (Example 2), with implications for transmission risk. Assuming no mosquito aggregation, mosquitoes are evenly spread across patches (Example 2 Panel B, no mosquito aggregation). If aggregation is weak (assuming high interference; Panel B, $m_{p}=0.9$), mosquitoes bias toward denser patches of hosts. With strong aggregation (low interference; Panel B, $m_{p}=0.1$), they concentrate heavily in those high host density patches.

Mosquito responses to host distribution result in variation in local biting pressure on competent hosts (Example 2C). When competent hosts cluster, bites become focused on competent hosts in dense patches as mosquito aggregation increases (low interference; $m_{p}=0.1$). When dead-end hosts strongly cluster (decay = 1), strong mosquito aggregation draws mosquitoes away from competent hosts in low-density patches, reducing bite rates. Under this scenario, with no or weak mosquito aggregation ($m_{p}=0.9$), however, there are more bites on competent hosts located in the patches where there are fewer dead-end hosts.

Local biting patterns, driven by host clustering and mosquito aggregation, ultimately determine the system-level outcomes, such as the overall proportion of bites directed at competent hosts across all patches (Example 2D). Clustering of competent hosts increases the overall proportion of blood meals taken on them under aggregation, with the lowest proportion occurring without mosquito aggregation. In contrast, clustering dead-end hosts reduces the proportion of bites on competent hosts under aggregation, while uniform mosquito distribution yields the highest proportion. We use these scenarios in the following sections to illustrate how varying assumptions about host and mosquito distributions may affect transmission under circumstances of increasing dead-end host density and of increasing fragmentation.

### Clustering of competent hosts and mosquito aggregation leads to increased transmission potential across a range of dead-end: competent host ratios when dead-end hosts are preferred

When assuming a low vector preference for competent hosts, transmission potential is similar to the baseline of a uniform distribution of hosts and mosquitoes, when competent hosts are clustered, across a range of dead-end to competent host ratios, if we assume that mosquitoes are uniformly distributed (Fig 3A, top row). However, if we assume mosquito aggregation, dilution occurs more rapidly when competent hosts are mildly clustered (decay = 0.2) compared to steep clustering (decay = 1). Except for relatively high dead-end to competent host ratios, if we assume strong mosquito aggregation and clustering of competent hosts, transmission potential will be larger than expected compared with the assumptions of uniform host and mosquito distributions.

**Fig 3:**
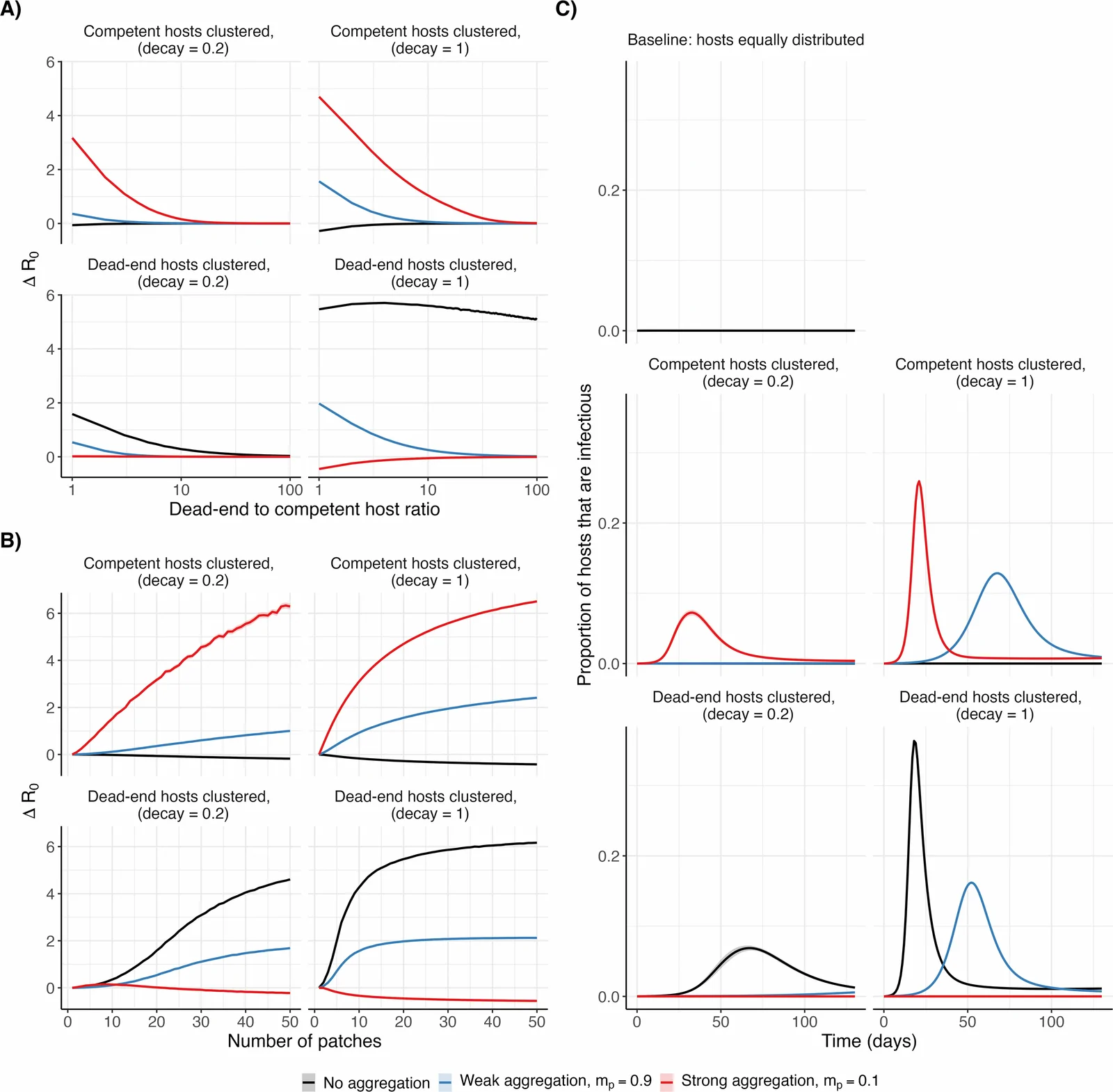
Heterogeneity in host distribution has the potential of amplifying or diluting transmission depending on mosquito aggregation. **(A)** The effect of the dead-end to competent host ratio on *R*_0_, given equal or clustered distributions of hosts in 20 patches with no, weak, or strong mosquito aggregation. **(B)** The effect on *R*_0_ of distributing hosts over an increasing number of patches. For A and B, each non-uniform host distribution scenario was replicated 200 times, with each replicate drawing a new multinomial host allocation across patches. The resulting *R*_0_ values were summarised and plotted as means with 95% CIs. **(C)** Dynamics of transmission in 20 patches. Parameters: $\eta_{x}=0.1$, $H_{x}=500$. Other parameters were kept constant (see Table 1). There were 100 replicate simulations per scenario, with 95% CIs on the proportion of infected competent hosts calculated at each time point. In all panels, 95% CIs are shown as shaded ribbons; where these are not visible, the intervals are narrower than the plotted line width.

Conversely, when dead-end hosts cluster (Fig 3A, bottom row), transmission potential is similar to baseline under strong mosquito aggregation. Without any mosquito aggregation, increasing dead-end host density only gradually reduces transmission, especially under steep clustering of dead-end hosts.

### Effects of distributing hosts over increasing number of patches depend on host clustering

Weak mosquito aggregation causes an increase in $\Delta R_{0}$ with increasing numbers of patches (Table 3). This effect is independent of which host type is clustered but is more pronounced if host clustering is high (Fig 3B). When mosquito aggregation is strong or absent, the effect of fragmentation is asymmetric and depends on which host type is clustered. Clustering competent hosts (Fig 3B, top row) results in a rapid increase in $\Delta R_{0}$ under strong mosquito aggregation and a gradual decline without aggregation. Whereas, clustering dead-end hosts (Fig 3B, bottom row) leads to a sharp rise in $\Delta R_{0}$ without mosquito aggregation and a slow decline under strong aggregation. In all scenarios, after a given number of patches, splitting the host community across more patches has little to no additional effect on transmission when compared to the baseline.

**Table 3:** Direction of change in *R*_0_ relative to baseline when distributing hosts over increasing numbers of patches, summarising Fig 3B.

Mosquito aggregation	Competent hosts clustered	Dead-end hosts clustered
No aggregation	$\Delta R_{0}$ decreases slightly	$\Delta R_{0}$ increases
Weak aggregation ($m_{p}=0.9$)	$\Delta R_{0}$ increases	$\Delta R_{0}$ increases
Strong aggregation ($m_{p}=0.1$)	$\Delta R_{0}$ increases	$\Delta R_{0}$ decreases slightly

### Effects of host and vector distributions on epidemic dynamics

In contrast to assuming a uniform host and vector distribution (Fig 3C, top row), which under the parameter values of Table 1 and low preference for competent hosts ($\eta_{x}=0.1$), does not produce an outbreak, strong mosquito aggregation under clustering of competent hosts causes the virus to spread faster through the susceptible host population, therefore producing the earliest and largest epidemic peaks (Fig 3C, middle row). As mosquito aggregation weakens, peak outbreak size reduces, with moderate transmission occurring under steep host clustering. Clustering dead-end hosts (Fig 3C, bottom row) produced the largest epidemic peak under uniform mosquito distribution, but the epidemic peak decreases as mosquito aggregation increases and mosquitoes are drawn toward patches with a relatively high density of dead-end hosts because of slower transmission rates.

In Fig 3A, when competent hosts are evenly distributed, while dead-end hosts are clustered (bottom right), some patches remain relatively competent-host dominated and can continue to support transmission, particularly when mosquitoes are not strongly aggregated in the dead-end-rich patches. In contrast, in the equal-distribution baseline, dead-end hosts are added to every patch, so dilution affects all competent-host patches simultaneously and the baseline *R*_0_ declines more rapidly. At low-to-moderate dead-end:competent host ratios, the baseline therefore declines faster than the aggregated scenario, causing $\Delta R_{0}$ to increase. At higher ratios, the baseline is already low and has less room to decline, while the aggregated scenario continues to lose transmission potential. $\Delta R_{0}$ therefore decreases again, producing the local maximum.

The results for the same scenarios, but assuming vectors prefer competent hosts ($\eta_{x}=0.9$), are presented in S2 File Fig 2. As shown in S2 File, Fig 2, at baseline there are already suitable conditions for transmission, and the dynamics and resulting timing and peak outbreak size depends on how hosts are distributed and the level of mosquito aggregation. When there is no or weak mosquito aggregation and dead-end hosts are clustered the results are similar to baseline (equal distribution of hosts and mosquitoes). When competent hosts are clustered, however, *R*_0_ is increased relative to baseline across a range of dead-end: competent host ratios (S2 File, Fig 2A) and the outbreak occurs earlier (S2 File, Fig 2C). The size of the outbreak is reduced when dead-end hosts are clustered and there is strong mosquito aggregation as, despite high preference for competent hosts, bites on them are reduced due to lower host relative availability in the highest density patches.

### Sensitivity analysis

Under the parameter ranges used (Table 1), spatial parameters (number of patches, host clustering decay rate, and interference) are the most variable drivers of *R*_0_, with their effects reversing direction dependent on host distribution (Fig 4). Specifically, interference is strongly negatively correlated with *R*_0_ when competent hosts are clustered, but strongly positively correlated with *R*_0_ when dead-end hosts are clustered. In contrast, the number of patches and the decay rate of host clustering are more weakly correlated with *R*_0_; they show a slight positive correlation when competent hosts cluster and a moderate negative correlation when dead-end hosts cluster. Among the other parameters, mosquito preference for competent hosts shows the strongest positive correlation with *R*_0_, while the dead-end-to-competent ratio shows the strongest negative correlation. Biting rate and transmission probabilities are moderately positively correlated with *R*_0_, whereas host recovery and mosquito mortality are more weakly negatively correlated.

**Fig 4:**
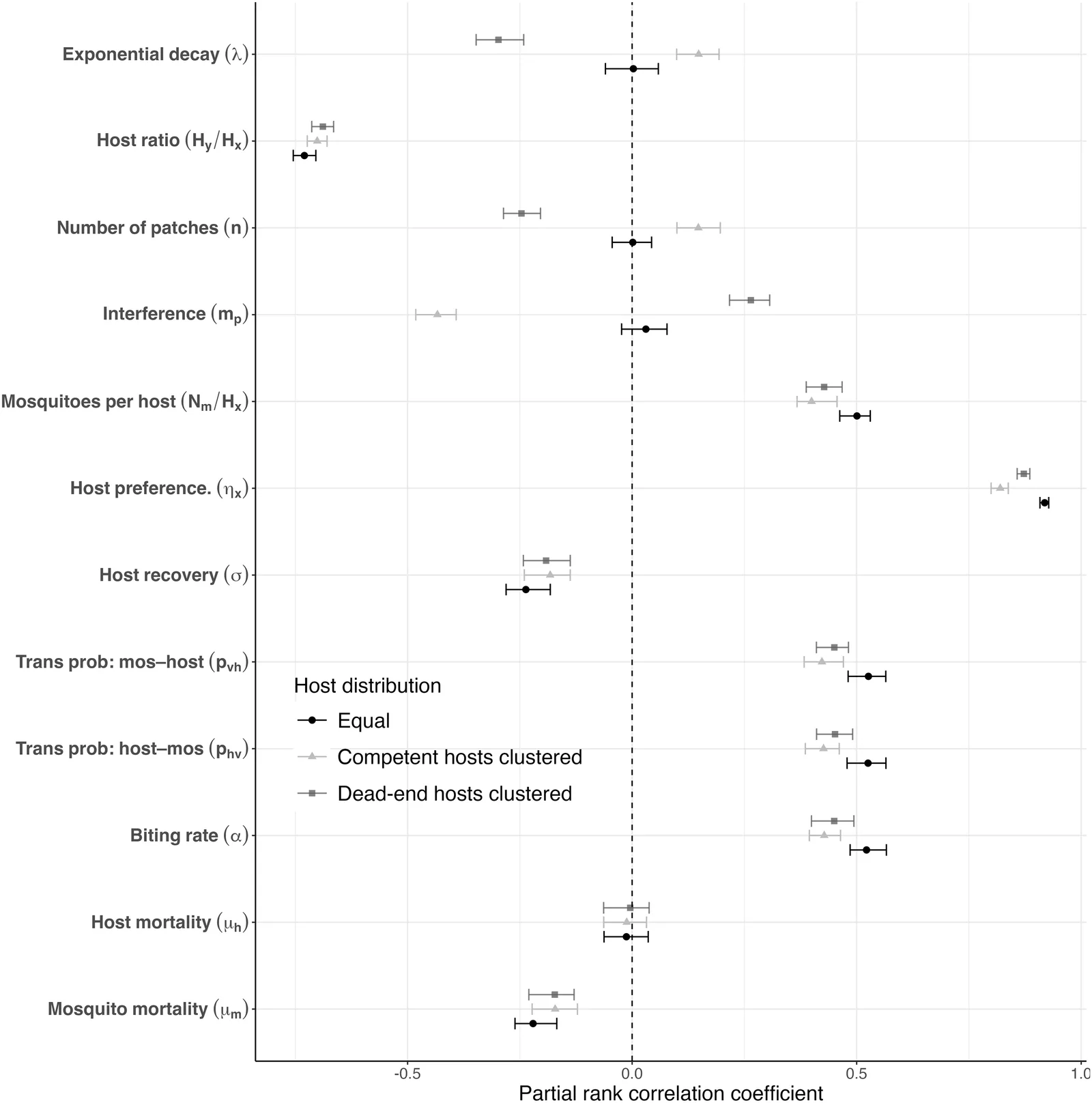
Partial rank correlation coefficients (PRCCs) between model parameters and *R*_0_ across different host distributions. Points are median PRCC estimates with the bars representing quantiles.

### Model limitations

Our work in this current study and that of [22] was motivated by the particular case of JEV in northwest Bangladesh, where cattle - dead-end hosts for the virus - outnumber pigs and are favoured by competent vector species [23,31]. We were interested to investigate potential causative pathways for zoonotic arbovirus maintenance under conditions such as this, where we would expect the high density of dead-end hosts to dilute and likely prevent transmission. As a consequence, to begin to explore the potential role of heterogeneity in biting due to long and short-range host seeking behaviour, here we focussed only on a restricted set of scenarios where mosquitoes were equally likely to visit any patch, long-range cues for patch choice was limited to carbon dioxide, and with patches hypothetically representing households where different numbers of livestock and poultry are kept. These assumptions were based on the relative pairwise distances between households in a village in relation to estimates of mosquito dispersal and assuming that hosts within a household could not be seen until mosquitoes arrive at a given household. Given that the larval habitats of the common JEV vector species are usually in rice paddies surrounding villages, they need to move between paddies and households between blood meals.

We accept that our modelling in this respect is a simplification and does not consider the circumstance where a mosquito is more likely to return to the household from which it previously fed, or more general spatial structuring with respect to mosquito dispersal between host-seeking, resting and oviposition sites. This may be explored in further work integrating metapopulation models with empirical data from field studies which are currently ongoing. Our framework, however, has provided us with a starting point and baseline understanding of how long- and short-range mosquito host seeking behaviour may influence transmission.

The modelling also does not account for seasonal mosquito dynamics or host movements and is limited to relatively simple scenarios involving only dead-end and competent host species and a single vector species, whereas in reality, transmission will likely involve different mosquito species of varying competence and host preference and hosts of varying competence. We also did not consider variations in host availability over time due to host movements and migrations, nor do we consider differences in host physical availability within a household, due to, for example, competent hosts being kept inside enclosed structures and dead-end hosts being easily accessible.

We accept that the host clustering was hypothetical to demonstrate the potential effects of non-uniform distribution, but realised host distributions among households may differ substantially from these arrangements and application to real-world context would involve the use of livestock and poultry census data from villages. We also only simulated a simple scenario of host fragmentation whereby a constant number of hosts were spread over an increasing number of patches. We acknowledge that our scenarios for *R*_0_ and epidemic dynamics are for a fixed vector:host ratio; with the assumption that any increases in this ratio would merely increase all values of *R*_0_ in the same way, thus not affecting the quantified relative change in *R*_0_ between each scenario and the baseline. Similarly, we did not include a mosquito exposed class to represent the extrinsic incubation period. Because only mosquitoes that survive this period become infectious, this simplification overestimates absolute *R*_0_. However, because the extrinsic incubation period does not depend on host distribution or mosquito aggregation, including it would scale *R*_0_ by the same factor across all scenarios, thereby reducing absolute values but leaving the relative comparisons that are the focus of this study unchanged. In this study, we have not attempted to use the framework to provide specific intervention recommendations or to model applied endpoints such as cattle seroprevalence or human spillover. Extending the framework to these applications would require additional dead-end-host exposure classes, and integration with field or surveillance data for a given arbovirus in a given transmission context.

Despite these limitations, these relatively simple scenarios were sufficient to outline the complexities that can arise when considering host-vector communities in spatially-explicit contexts, and to determine the potential effects of long- and short-range host seeking on transmission without creating an overly complex model.

## Discussion

Zoonotic mosquito-borne viruses are often transmitted within and between communities of multiple host and vector species. Here, our work contributes to furthering understanding of the potential impacts of host community composition on vector host choice and pathogen transmission. We introduced host clustering to show how the same host community can produce different transmission potentials depending on their relative distribution across patches and mosquito aggregation behaviour. We draw attention to the importance of host community structure and distribution across the landscape and the host-seeking responses of mosquitoes. Evaluation of transmission risk requires consideration of not only vector host preference and host abundance, but also the spatial context in which host-vector interactions occur.

To follow from our previous work [22], we aimed to demonstrate the potential effects of considering biological complexity introduced by mosquito short and long-range host-seeking on host choice and therefore transmission. We focussed particularly on how transmission may be maintained even under conditions of low vector preference for competent hosts and a dominance of dead-end hosts in the community. Our study highlights that local-scale (e.g., 100s m) variation in host community composition may substantially affect the force of infection and lead to local-scale heterogeneity in transmission patterns. The modelling assists in thinking through circumstances that can lead to both local-scale heterogeneity and sustained transmission in regions where this may not be expected from more regional analyses.

Similar concerns were raised by Perkins *et. al.* [51], who showed that heterogeneity in mosquito movement and the spatial distribution of mosquitoes and hosts generates characteristic spatial scales of transmission, and failing to account for these scales can mislead inference about epidemic dynamics and critical vaccination thresholds. Complementing this, our work adds biological detail by explicitly separating host types and incorporating biological mechanisms that generate heterogeneity in mosquito biting. At long-range, we modelled host-seeking behaviour using principles from classic predator–prey foraging theory, where mosquitoes, like predators, aggregate in response to host density but are subject to interference in resource-dense patches [34,38]. At short-range, variation in biting arises from host traits such as body size, which affect attractiveness and contact rates.

The scale-dependent nature of these vector–host interactions has important implications for pathogen surveillance. Local bite patterns can differ substantially from system-wide averages, therefore, depending on the sampling design, bloodmeal surveys pooled over large areas may give a different picture of vector–host contact than fine-scale surveys, potentially obscuring local transmission risks. Recent reviews of West Nile virus models show that, despite evidence for spatial heterogeneity and recommendations to incorporate host heterogeneity [52], most mechanistic models assume homogeneity [42]. While some models include multiple host species with species-specific infection parameters or vector biting preferences, few account for imperfect mixing or spatial heterogeneity [42].

Heterogeneity in host and vector distribution can arise from human-driven and environmental changes, such as agricultural intensification, urbanisation, irrigation projects, and livestock management, which can create locally clustered host populations or modify mosquito breeding habitats. For example, Jones *et. al.* [53] hypothesised that large-scale irrigation in southern Malawi could fragment the landscape, potentially creating new human–animal–mosquito interactions and increasing exposure to a broader diversity of vector species. Our model provides a basic framework for quantitatively exploring the transmission risks that might emerge from such landscape modifications before they are implemented.

Moderate clustering of competent hosts could account for the persistence of virus transmission in contexts where dead-end hosts are dominant. Our model predicts that more intensive farming of competent hosts, kept separate from other vertebrate species, likely creates transmission hotspots if local vectors strongly aggregate in response to host density. While this finding may be logical in contexts where competent hosts are dominant, without the need for our more nuanced model, our results also explain the possibility of transmission in potentially unexpected contexts. For JEV, our work here builds on our findings, in our previous work, that vector host species fidelity (repeated biting on the same host type) could help to explain transmission where dead-end hosts (cattle) are dominant in the community and are preferred by vector species [22]. For example, in northwest Bangladesh where human JE cases occur, the cattle (dead-end hosts) to pig (competent hosts) ratio is > 140: 1 [31]. However, poultry, which also produce sufficient viremia to infect mosquitoes, are relatively common here, and the majority of pigs that are present are kept in separate, mobile herds [54]. The spatial structuring of poultry flocks and pig herds relative to cattle may explain the possibility of maintained JEV transmission in this region, despite the relatively high density of dead-end hosts in this environment. This hypothesis would of course require empirical field studies to gather evidence to support or refute.

Our relatively simple scenarios were sufficient to outline the complexities that can arise when considering host-vector communities in spatially-explicit contexts. Our next steps will be to apply our theoretical findings to analyses involving field data to quantify zoonotic mosquito-borne virus transmission in contexts like northwest Bangladesh, where dead-end hosts are dominant species in the community.

## Supporting information

S1 File Supplementary methods: Derivation of the basic reproduction number and estimation of mosquito aggregation from catch data. Fig. 1 Aggregation of female mosquitoes across households in Bangladesh.
(PDF)

S2 File Additional results: Fig 1 Effect of increasing number of patches on host distribution. Fig 2 Host clustering and mosquito aggregation influence mosquito host choice. As per Fig 2. in main manuscript, but assuming high vector host preference for competent hosts.
(PDF)
