# Supplementary material for: The impacts of host community composition, clustering and vector host choice on zoonotic mosquito-borne virus transmission
Source: PLoS Negl Trop Dis. 2026 Oct 22;20(10):e0014758. doi: 10.1371/journal.pntd.0014758 (PMC13719057; doi:10.1371/journal.pntd.0014758)
Supplement: Supplementary file 1 [file pntd.0014758.s001.pdf]

Supplementary File 1

Supplementary methods

Basic reproduction number

The basic reproduction number R_0, defined as the expected number of secondary cases produced by a typical infected individual in a wholly susceptible population. Mathematically, R_0 corresponds to the dominant eigenvalue of the next-generation matrix (NGM) FV^{-1}, where the matrix F is the rate at which new infections are produced and V is the rate at which individuals enter or leave the infection compartments [1].

$$
\begin{aligned}
\frac{dS_x}{dt} &= \mu_x H_x - \beta_{vh}\rho_x I_v \frac{S_x}{H_x} - \mu_x S_x \\
\frac{dI_x}{dt} &= \beta_{vh}\rho_x I_v \frac{S_x}{H_x} - (\mu_x + \sigma) I_x \\
\frac{dR_x}{dt} &= \sigma I_x - \mu_x R_x \\
\frac{dS_v}{dt} &= \mu_v N_v - \beta_{hv}\rho_x \frac{I_x}{H_x} S_v - \mu_v S_v \\
\frac{dI_v}{dt} &= \beta_{hv}\rho_x \frac{I_x}{H_x} S_v - \mu_v I_v
\end{aligned} \tag{1}
$$

For the system in Equation 1, the disease-free equilibrium (DFE) is $x_0 = (S_x, I_x, S_v, I_v) = (H_x, 0, N_v, 0)$. The vectors f and v are defined as:

$$
f = \begin{bmatrix} \alpha\rho_x\beta_{vh} I_v \frac{S_x}{H_x} \\ \alpha\rho_x\beta_{hv} \frac{I_x}{H_x} S_v \end{bmatrix} \quad \text{and} \quad v = \begin{bmatrix} (\mu_x + \sigma) I_x \\ \mu_v I_v \end{bmatrix}.
$$

The matrices F and V are derived by evaluating the Jacobians of f and v at DFE:

$$
F = \begin{bmatrix} 0 & \alpha\rho_x\beta_{vh} \\ \alpha\rho_x\beta_{hv}\frac{N_v}{H_x} & 0 \end{bmatrix} \quad \text{and} \quad V = \begin{bmatrix} (\mu_x + \sigma) & 0 \\ 0 & \mu_v \end{bmatrix}.
$$

$$
V^{-1} = \begin{bmatrix} \frac{1}{(\mu_x+\sigma)} & 0 \\ 0 & \frac{1}{\mu_v} \end{bmatrix}.
$$

The next-generation matrix FV^{-1} becomes:

$$
FV^{-1} = \begin{bmatrix} 0 & \frac{\alpha\rho_x\beta_{vh}}{\mu_v} \\ \frac{\alpha\rho_x\beta_{hv}N_v}{(\mu_x+\sigma)H_x} & 0 \end{bmatrix} \tag{2}
$$

The largest eigenvalue of FV^{-1} is therefore

$$
R_0 = \sqrt{\underbrace{\frac{\alpha\rho_x\beta_{vh}}{\mu_v}}_{\text{vector to host species } x} \cdot \underbrace{\frac{\alpha\rho_x\beta_{hv}}{(\mu_x+\sigma)}\frac{N_v}{H_x}}_{\text{host species } x \text{ to vector}}}. \tag{3}
$$

In Equation 3, the first term represents the number of secondary host species infections x caused by an infected vector, and the second term represents the number of secondary vector infections caused by an infected host species x.

$$\begin{aligned}
\frac{dS_{x,i}}{dt} &= \mu_x H_{x,i} - \beta_{vh}\rho_x\big(\delta_{v,i}I_v\big)\frac{S_{x,i}}{H_{x,i}} - \mu_x S_{x,i} \\
\frac{dI_{x,i}}{dt} &= \beta_{vh}\rho_x\big(\delta_{v,i}I_v\big)\frac{S_{x,i}}{H_{x,i}} - (\mu_x + \sigma)I_{x,i} \\
\frac{dR_{x,i}}{dt} &= \sigma I_{x,i} - \mu_x R_{x,i} \\
\frac{dS_v}{dt} &= \mu_v N_v - \beta_{hv}\rho_x \sum_i^n \frac{I_{x,i}}{H_{x,i}}\big(\delta_{v,i}S_v\big) - \mu_v S_v \\
\frac{dI_v}{dt} &= \beta_{hv}\rho_x \sum_i^n \frac{I_{x,i}}{H_{x,i}}\big(\delta_{v,i}S_v\big) - \mu_v I_v
\end{aligned} \tag{4}$$

In the system described in Equation 4, the matrix F is an $(n+1)\times(n+1)$ matrix, where each element F_{ij} represents the rate at which new infections in population i are caused by infected individuals in population j. The populations are ordered as host infections in patches 1 to n, followed by a single vector population. Since transmission occurs only between susceptible hosts in a patch and visiting infected vectors, and between infected hosts and visiting susceptible vectors, all elements of F are zero, except for:

$$F_{k,n+1} = \beta_{vh}\rho_x\delta_{m,k},$$

and

$$F_{n+1,k} = \frac{\beta_{hv}\rho_x\delta_{m,k}N_v}{H_{x,k}},$$

for $k = 1, 2, \cdots, n$. To see this, we derive the NGM for Equation 4, starting with defining the vectors f.

$$f = \begin{pmatrix}
\beta_{vh}\rho_x\delta_{m,1}I_v\frac{S_{x,1}}{H_{x,1}} \\
\beta_{vh}\rho_x\delta_{m,2}I_v\frac{S_{x,2}}{H_{x,2}} \\
\vdots \\
\beta_{vh}\rho_x\delta_{m,n}I_v\frac{S_{x,n}}{H_{x,n}} \\
\beta_{hv}\rho_x[\delta_{m,1}S_v\frac{I_{x,1}}{H_{x,1}} + \delta_{m,2}S_v\frac{I_{x,2}}{H_{x,2}} + \cdots + \delta_{m,n}S_v\frac{I_{x,n}}{H_{x,n}}]
\end{pmatrix}
\begin{matrix} \text{Patch 1} \\ \text{Patch 2} \\ \\ \text{Patch n} \\ \text{Mosquitoes} \end{matrix}$$

$$\begin{matrix} & \text{Patch 1} & \text{Patch 2} & \cdots & \text{Patch n} & \text{Mosquitoes} & \end{matrix}$$

$$F = \begin{pmatrix}
0 & 0 & \cdots & 0 & \beta_{vh}\rho_x\delta_{m,1} \\
0 & 0 & \cdots & 0 & \beta_{vh}\rho_x\delta_{m,2} \\
\vdots & \vdots & \ddots & \vdots & \vdots \\
0 & 0 & \cdots & 0 & \beta_{vh}\rho_x\delta_{m,n} \\
\frac{\beta_{hv}\rho_x\delta_{m,1}N_v}{H_{x,1}} & \frac{\beta_{hv}\rho_x\delta_{m,2}N_v}{H_{x,2}} & \cdots & \frac{\beta_{hv}\rho_x\delta_{m,n}N_v}{H_{x,n}} & 0
\end{pmatrix}
\begin{matrix} \text{Patch 1} \\ \text{Patch 2} \\ \vdots \\ \text{Patch } n \\ \text{Mosquitoes} \end{matrix}$$

$$V^{-1} = \begin{array}{c} \begin{matrix} \text{Patch 1} & \text{Patch 2} & \cdots & \text{Patch n} & \text{Mosquitoes} \end{matrix} \\ \begin{pmatrix} \frac{1}{(\mu_x+\sigma)} & 0 & \cdots & 0 & 0 \\ 0 & \frac{1}{(\mu_x+\sigma)} & \cdots & 0 & 0 \\ \vdots & \vdots & \ddots & \vdots & \vdots \\ 0 & 0 & \cdots & \frac{1}{(\mu_x+\sigma)} & 0 \\ 0 & 0 & 0 & 0 & \frac{1}{\mu_v} \end{pmatrix} \end{array} \begin{matrix} \text{Patch 1} \\ \text{Patch 2} \\ \vdots \\ \text{Patch } n \\ \text{Mosquitoes} \end{matrix}$$

$$FV^{-1} = K_n = \begin{array}{c} \begin{matrix} \text{Patch 1} & \text{Patch 2} & \cdots & \text{Patch n} & \text{Mosquitoes} \end{matrix} \\ \begin{pmatrix} 0 & 0 & \cdots & 0 & \frac{\beta_{vh}\rho_x\delta_{m,1}}{\mu_v} \\ 0 & 0 & \cdots & 0 & \frac{\beta_{vh}\rho_x\delta_{m,2}}{\mu_v} \\ \vdots & \vdots & \ddots & \vdots & \vdots \\ 0 & 0 & \cdots & 0 & \frac{\beta_{vh}\rho_x\delta_{m,n}}{\mu_v} \\ \frac{\beta_{hv}\rho_x\delta_{m,1}N_v}{(\mu_x+\sigma)H_{x,1}} & \frac{\beta_{hv}\rho_x\delta_{m,2}N_v}{(\mu_x+\sigma)H_{x,2}} & \cdots & \frac{\beta_{hv}\rho_x\delta_{m,n}N_v}{(\mu_x+\sigma)H_{x,n}} & 0 \end{pmatrix} \end{array} \begin{matrix} \text{Patch 1} \\ \text{Patch 2} \\ \vdots \\ \text{Patch } n \\ \text{Mosquitoes} \end{matrix} \tag{5}$$

Special cases: For the special case in which host are in a single population, that is $n = 1$, the NGM matrix K_n in Equation 5 will reduce to the matrix in Equation 2, where $\delta_{m,1} = 1$. For $n = 2$, the NGM matrix K_n in Equation 5 will reduce to a 3 by 3 matrix K_2 given by

$$K_2 = \begin{array}{c} \begin{matrix} \text{Patch 1} & \text{Patch 2} & \text{Mosquitoes} \end{matrix} \\ \begin{pmatrix} 0 & 0 & \frac{\beta_{vh}\rho_x\delta_{m,1}}{\mu_v} \\ 0 & 0 & \frac{\beta_{vh}\rho_x\delta_{m,2}}{\mu_v} \\ \frac{\beta_{hv}\rho_x\delta_{m,1}N_v}{(\mu_x+\sigma)H_{x,1}} & \frac{\beta_{hv}\rho_x\delta_{m,2}N_v}{(\mu_x+\sigma)H_{x,2}} & 0 \end{pmatrix} \end{array} \begin{matrix} \text{Patch 1} \\ \text{Patch 2} \\ \text{Mosquitoes} \end{matrix} .$$

It is easy to see that the largest eigenvalue of K_2 is

$$R_0^{K_2} = \sqrt{\left(\frac{\beta_{vh}\rho_x\delta_{m,1}}{\mu_v} \cdot \frac{\beta_{hv}\rho_x\delta_{m,1}N_v}{(\mu_x+\sigma)H_{x,1}}\right) + \left(\frac{\beta_{vh}\rho_x\delta_{m,2}}{\mu_v} \cdot \frac{\beta_{hv}\rho_x\delta_{m,2}N_v}{(\mu_x+\sigma)H_{x,2}}\right)}.$$

Similarly,

$$R_0^{K_3} = \sqrt{\begin{array}{l} \left(\frac{\beta_{vh}\rho_x\delta_{m,1}}{\mu_v} \cdot \frac{\beta_{hv}\rho_x\delta_{m,1}N_v}{(\mu_x+\sigma)H_{x,1}}\right) + \\ \left(\frac{\beta_{vh}\rho_x\delta_{m,2}}{\mu_v} \cdot \frac{\beta_{hv}\rho_x\delta_{m,2}N_v}{(\mu_x+\sigma)H_{x,2}}\right) + \\ \left(\frac{\beta_{vh}\rho_x\delta_{m,3}}{\mu_v} \cdot \frac{\beta_{hv}\rho_x\delta_{m,3}N_v}{(\mu_x+\sigma)H_{x,3}}\right) \end{array}}$$

Consequently,

$$R_0 = R_0^{K_n} = \sqrt{\sum_i^n \left(\frac{\beta_{vh}\rho_x\delta_{v,i}}{\mu_v} \cdot \frac{\beta_{hv}\rho_x\delta_{v,i}N_v}{(\mu_x+\sigma)H_{x,i}}\right)}. \tag{6}$$

Estimating mosquito aggregation from catch data

We fitted the Sutherland model to household mosquito catch data from Bangladesh [2] using log-normal and negative binomial likelihoods. The log-normal model provided a better fit (AIC = 312) than the negative binomial (AIC = 1405). Both models fits the aggregation pattern better than the unrealistic assumptions of strict proportional to host density ($m_p = 1$) or uniform distribution (Figure 1).

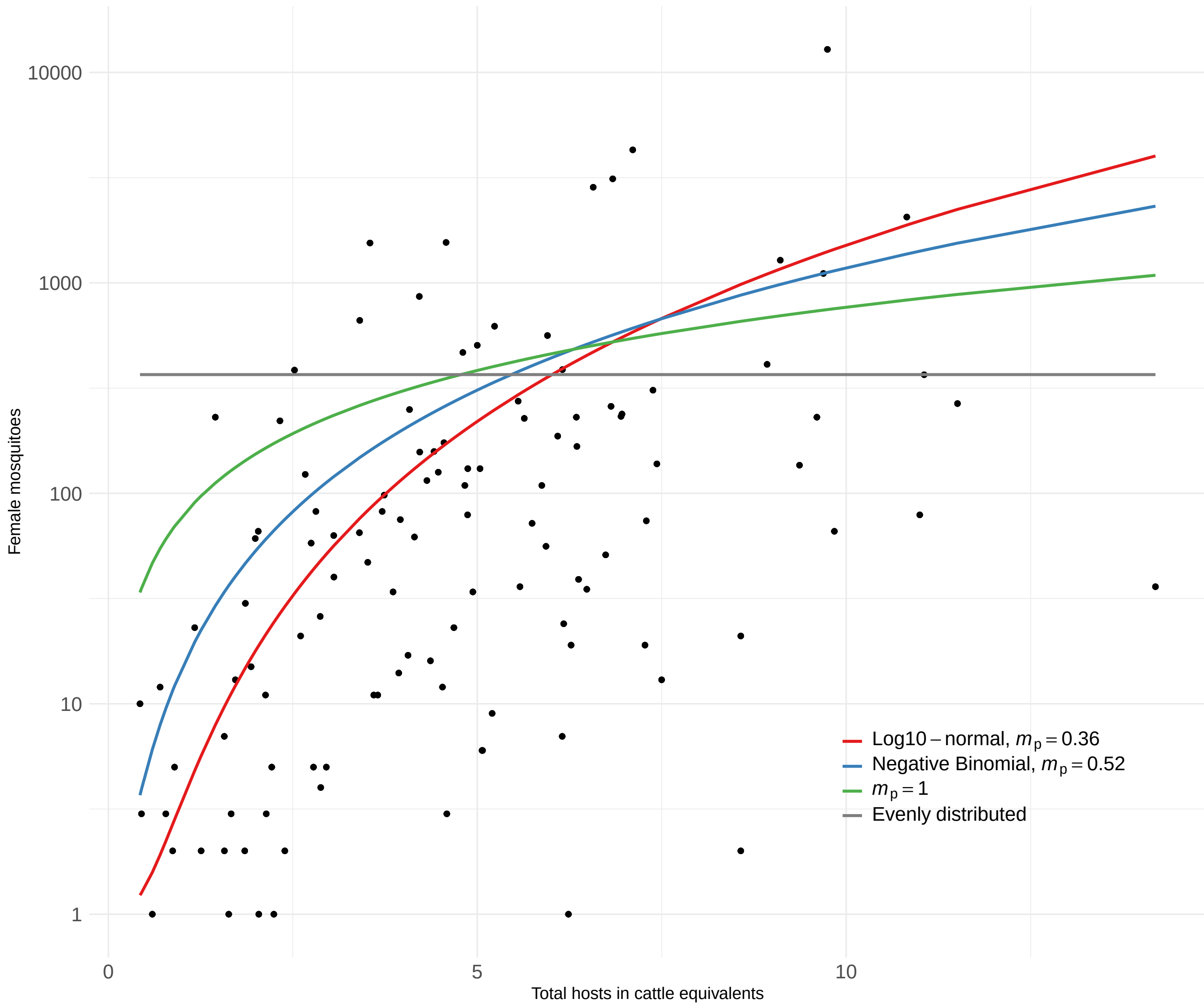

Figure 1: Aggregation of female mosquitoes across households in Bangladesh. Points represent observed mosquito counts, and lines show model predictions as a function of cattle-equivalent hosts. Coloured lines represent fitted models; gray and green lines show extreme assumptions: uniform mosquito distribution and a distribution proportional to host density ($m_p = 1$).

References

1. Diekmann O, Heesterbeek JAP, Metz JAJ. On the definition and the computation of the basic reproduction ratio R 0 in models for infectious diseases in heterogeneous populations. Journal of Mathematical Biology. 1990;28(4). doi:10.1007/bf00178324.

2. Lord JS, Al-Amin HM, Chakma S, Alam MS, Gurley ES, Pulliam JRC. Sampling design influences the observed dominance of Culex tritaeniorhynchus: considerations for future studies of Japanese encephalitis virus transmission. PLOS Neglected Tropical Diseases. 2016;10(1):e0004249. doi:10.1371/journal.pntd.0004249.
