# Supplementary material for: The impacts of host community composition, clustering and vector host choice on zoonotic mosquito-borne virus transmission
Source: PLoS Negl Trop Dis. 2026 Oct 22;20(10):e0014758. doi: 10.1371/journal.pntd.0014758 (PMC13719057; doi:10.1371/journal.pntd.0014758)
Supplement: Supplementary file 2 [file pntd.0014758.s002.pdf]

Supplementary File 2

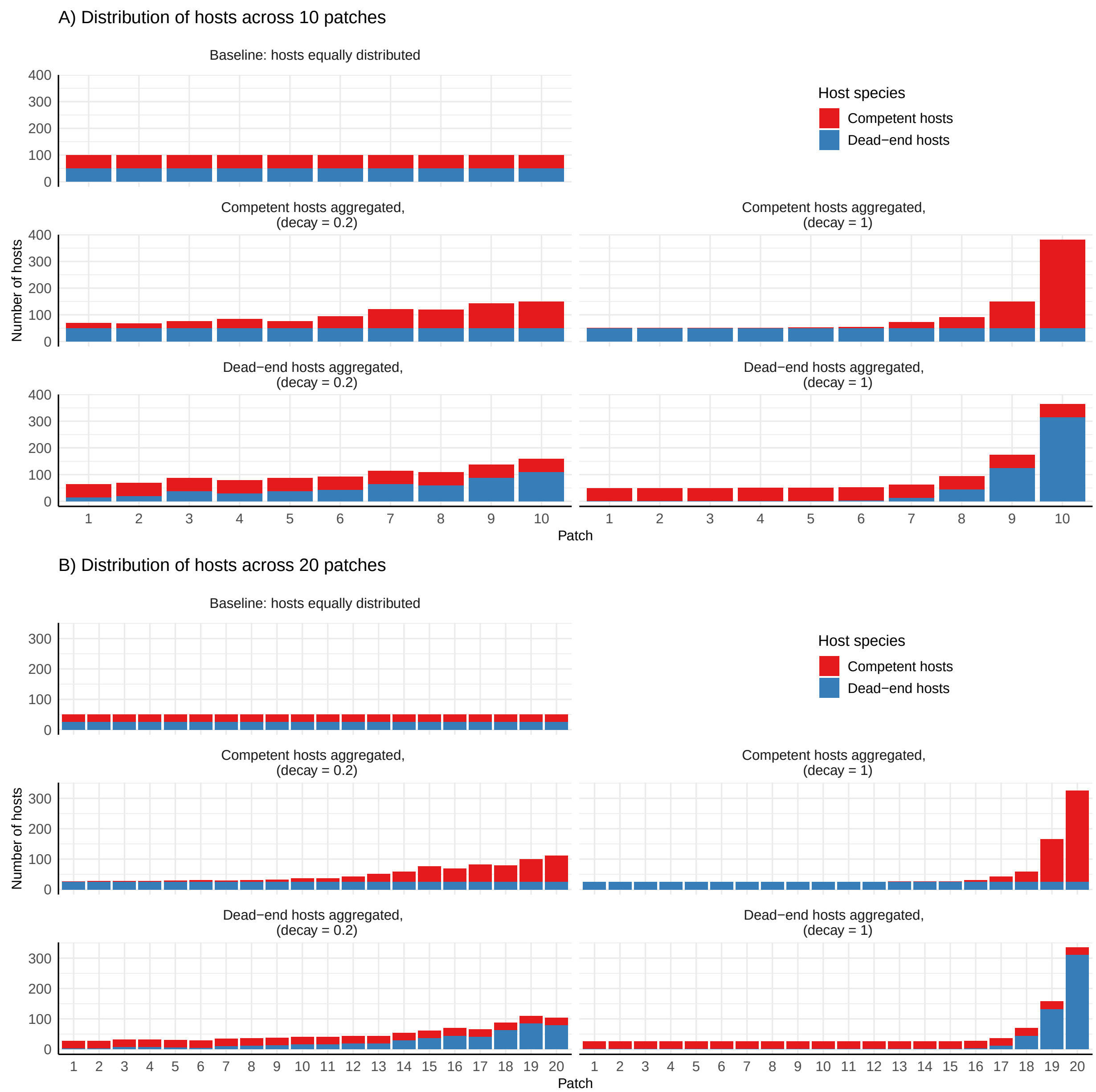

Fig 1. Effect of increasing number of patches on host distribution. When the total number of hosts is constant, distributing hosts over a greater number of patches means that: i) there are overall fewer hosts in each patch; ii) when competent hosts are aggregated there are a greater number of patches supporting only dead-end hosts and *vice versa* for when dead-end hosts are aggregated.

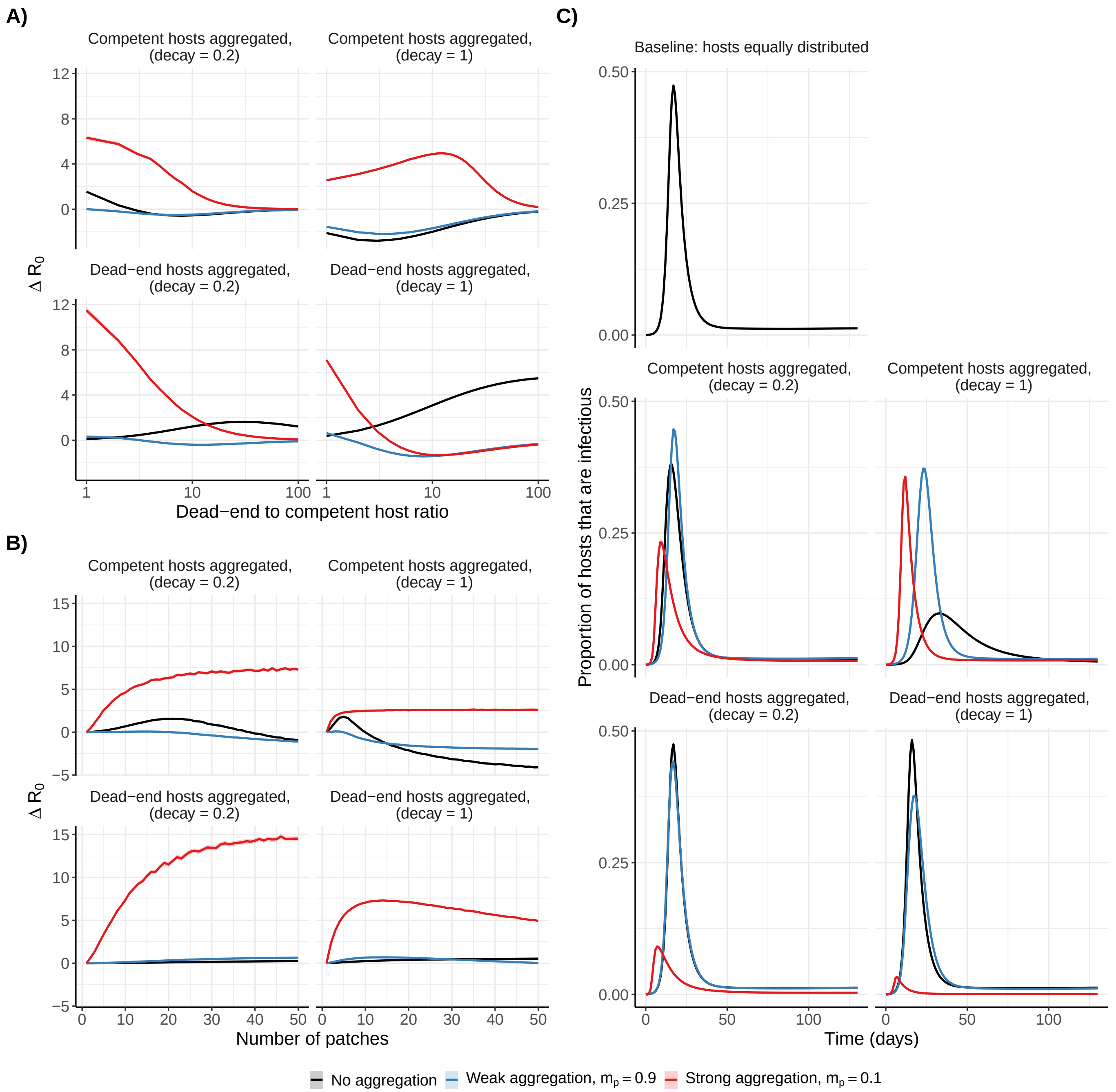

Fig 2. Host clustering and mosquito aggregation influence mosquito host choice.. As per Fig. 2. in main manuscript, but assuming high vector host preference for competent hosts. (A) The effect of the dead-end to competent host ratio on R_0, given weak or strong host clustering in 20 patches with no, weak, or strong mosquito aggregation. (B) The effect on R_0 of distributing hosts over an increasing number of patches. For A and B, each non-uniform host distribution scenario was replicated 200 times, with each replicate drawing a new multinomial host allocation across patches. The resulting R_0 values were summarised and plotted as means with 95% CIs, shown as shaded ribbons. (C) Dynamics of transmission in 20 patches. Parameters: $\eta_x = 0.9$, $H_x = 500$. Other parameters were kept constant (see Table 1 of main manuscript). There were 100 replicate simulations per scenario, with 95% CIs on the proportion of infected competent hosts calculated at each time point and displayed as ribbons.
